# Epidemiological, clinical, and genotypic characteristics of pediatric *Mycoplasma pneumoniae* infections: an 8-year survey in Suzhou, China in the pre- and post-COVID-19 eras

**DOI:** 10.3389/fmicb.2024.1483152

**Published:** 2024-10-15

**Authors:** Lina Xu, Pengli Wang, Yufeng Wang, Bingjie Liu, Xuena Xu, Quying Yang, Chunyan Gao, Huiquan Sun, Yuejuan Xu, Qiuyan Xu, Chuangli Hao, Wujun Jiang

**Affiliations:** ^1^Department of Respiratory Medicine, Children’s Hospital of Soochow University, Suzhou, China; ^2^Department of Respiratory Medicine, Children’s Hospital of Wujiang District, Suzhou, China; ^3^Department of Pediatrics, Suzhou Research Center of Medical School, Suzhou Hospital, Affiliated Hospital of Medical School, Nanjing University, Suzhou, China

**Keywords:** children, *Mycoplasma pneumoniae*, genotype, COVID-19, China

## Abstract

**Objective:**

This study examines the epidemiology of *Mycoplasma pneumoniae* (*M. pneumoniae*) infections among children in Suzhou, China, during various pandemic phases. The goal is to discern evolving epidemic trends and to furnish robust evidence for clinical diagnosis and treatment.

**Methods:**

From January 1, 2016, to December 31, 2023, 113,625 consecutive patients with respiratory infections from three hospitals in Suzhou, China (Children’s Hospital of Soochow University, Children’s Hospital of Wujiang District, and Affiliated Suzhou Hospital of Nanjing University Medical School), were retrospectively enrolled in a surveillance study. Additionally, in 2023, children hospitalized with *M. pneumoniae* pneumonia at the Children’s Hospital of Soochow University were tested for genotype (P1 gene typing, SNP genotyping) and macrolide resistance in their bronchoalveolar lavage fluid.

**Results:**

From 2016 to 2023, the *M. pneumoniae* positive detection rate among pediatric respiratory infections fell from a pre-pandemic 21.1% to pandemic lows, then surged to 45.3% post-pandemic. Before the pandemic, peak *M. pneumoniae* infection rates occurred in summer, followed by autumn. Post-pandemic, the highest peak rates were in autumn. Peak *M. pneumoniae* detection rates occurred in 2019 and 2023, with a notable increase in children aged 6 and older in 2023. In this study, 200 *M. pneumoniae*-positive bronchoalveolar lavage fluid (BALF) cases in 2023 were randomly selected and analyzed for P1 genotype and SNP genotype. Among 156 cases, 81.4% were P1 genotype and 18.6% were P2 genotype. The proportion of severe *M. pneumoniae* pneumonia with the P1 type was significantly higher than that with the P2 type (*p* < 0.05). Of the 192 samples analyzed, 11 SNP genotypes were identified, with SNP-27 predominating (36.5%), followed by SNP-0 (21.4%), SNP-11 (18.8%), and SNP-34 (17.7%). Of the 192 BALF specimens, 97.3% exhibited macrolide resistance mutations, with A2063G mutations at 96.17%. The mutation rates for the 23S rRNA 2064 and 2,617 were 1.6 and 1.0%, respectively.

**Conclusion:**

Post-COVID-19 in Suzhou, China, *M. pneumoniae* infection patterns shifted significantly, with initial NPIs-induced declines followed by a sharp rise in cases, especially impacting school-age children. This trend underscores the importance of ongoing epidemiological surveillance and the development of strategic public health responses.

## Introduction

*Mycoplasma pneumoniae* is one of the leading causes of community-acquired pneumonia (CAP) in children and adults (Waites et al., 2017). *M. pneumoniae* has a global distribution and tends to resurge every few years. In adults across Europe, *M. pneumoniae* pneumonia prevalence ranges from 4 to 8% in the off-season, and peaks at 20–40% in the on-season (Bajantri et al., 2018). In China, *M. pneumoniae* infections constitute 10–40% of CAP cases in children, imposing substantial economic and social burdens (Waites et al., 2017; He et al., 2013).

During the COVID-19 pandemic, many countries implemented non-pharmaceutical interventions (NPIs) to suppress the propagation of pathogens (Cowling et al., 2020). The trajectory of infectious diseases shifted during the COVID-19 pandemic, with a notable decrease in respiratory infections. Early 2020 data from several countries suggested that NPIs contributed to a reduced incidence of *M. pneumoniae* (Oster et al., 2020; Haapanen et al., 2021). However, following the pandemic’s subsidence, there was a swift rise in respiratory infections, particularly among children, with illness durations extending beyond the norm (Edens et al., 2024; Meyer Sauteur et al., 2022a; Cason et al., 2022). The commentary by Meyer Sauteur et al. (2022a), published in *The Lancet Microbe*, highlights the anticipated resurgence of *M. pneumoniae* and its significant anticipated impact following COVID-19. Despite the lifting of COVID-19 restrictions, China’s epidemiological data on *M. pneumoniae* remain scarce (Sauteur and Beeton, 2024). In 2023, China saw a large-scale outbreak of *M. pneumoniae* in children, occurring earlier than in Europe and marked by greater severity and complications than prior outbreaks.

Although multiple studies have detailed the epidemiological and genetic profiles of *M. pneumoniae* infections in China prior to the COVID-19 pandemic, understanding of the post-pandemic genotypes and resistance patterns remains limited (Zhao et al., 2019; Rodman et al., 2021). China’s course of the COVID-19 pandemic and its approach to NPIs are distinct compared to other countries. In the first 3 years of the pandemic, China enforced strict NPIs, but the relaxation by the end of 2022 resulted in a significant increase in COVID-19 cases across the country. Thus, comprehending the epidemiological trajectory of *M. pneumoniae* in China from the start of the COVID-19 pandemic is essential. It is also critical to examine how NPIs, population immunity, and SARS-CoV-2 infections, including genotype and antibiotic resistance, interact to influence *M. pneumoniae* epidemic patterns. Further investigation of these interactions will enhance our ability to predict and prepare for future *M. pneumoniae* outbreaks, offering a scientific foundation for establishing effective prevention and control strategies.

This study examines the epidemiology of *M. pneumoniae* infections among children in Suzhou, China, across various pandemic phases, including genotyping of circulating strains and detection of resistance genes. By conducting a detailed analysis of *M. pneumoniae* infections in Suzhou’s pediatric population, this study aims to clarify shifts in epidemic trends and inform more effective clinical diagnosis and treatment strategies.

## Methods

### Ethics statement

The protocol of this study was approved by the Ethics Committee of the Children's Hospital of Soochow University (ethical approval no. 2023CS116) and was in line with the Declaration of Helsinki (as revised in 2013).

### Study population

From January 1, 2016, to December 31, 2023, our surveillance study retrospectively enrolled a total of 113,625 patients with respiratory infections from three hospitals in Suzhou, China: the Children’s Hospital of Soochow University, Children’s Hospital of Wujiang District, and Affiliated Suzhou Hospital of Nanjing University Medical School. Among them, there were a total of 82,427 pediatric cases from the Children’s Hospital of Soochow University.

In this study, 200 children with *M. pneumoniae* from the Children’s Hospital of Soochow University were enrolled between June and December 2023. Notably, this testing was exclusive to this hospital and not extended to other participating hospitals. These patients, part of the respiratory infection surveillance from 2016 to 2023, underwent P1 gene typing, multi-site single nucleotide polymorphism (SNP) genotyping, and macrolide resistance testing on BALF samples. For each, we documented general information, clinical signs, BALF characteristics, and radiological findings. Inclusion criteria were as follows: The inclusion criteria were: (I) meeting the diagnostic criteria for CAP (Harris et al., 2011; Rose et al., 2020); (II) selection of pediatric patients under 18 years of age; (III) obtaining BALF by bronchoscopy in eligible patients; (IV) BALF testing positive for *M. pneumoniae* DNA. Exclusion criteria included: (I) presence of tuberculosis, interstitial lung disease, pulmonary eosinophilic infiltration, or pulmonary vasculitis; (II) incomplete clinical data.

### Laboratory assays

The collected respiratory specimens were subjected to nucleic acid (DNA) testing for *M. pneumoniae* (Thermo Scientific™ KingFisher™ Flex Magnetic Particle Processors, Thermo Fisher). All atypical pathogens were tested with the use of the commercial real-time PCR based kits (Multiplex Combined Real-time PCR Detection Kit for Respiratory Viruses, Multiplex Combined Real-time PCR Detection Kit for Respiratory Bacteria, Jiangsu Uninovo Biological Technology Co. Ltd., China).

### P1 gene typing

P1 gene sequences from *M. pneumoniae*–positive specimens were typed using nested PCR and restriction fragment length polymorphism (RFLP), as previously described. The RepMP2/3 and RepMP4 repetitive regions were amplified concurrently, and the PCR products from type 2 specimens were sequenced to identify type 2 variants (Hung et al., 2021; Pereyre et al., 2007).

### Multisite SNP genotyping method for *M. pneumoniae*

A novel SNP genotyping method for *M. pneumoniae* was developed utilizing matrix-assisted laser desorption/ionization time-of-flight mass spectrometry (MALDI-TOF MS) (Zhao et al., 2021). The detection limit of this method for nucleic acids was 10^2^–10^3^ copies/reaction. Six SNP site-based genotypings were simultaneously detectable using a multiplex PCR and mass probe assay, as detailed in Supplementary Table S1.

### Macrolide resistance-associated mutations

Mutations associated with macrolide resistance, specifically at positions 2063, 2064, and 2,617 in the 23S rRNA gene of *M. pneumoniae*, were identified as previously reported (Lin et al., 2010).

### General *M. pneumoniae* pneumonia and severe *M. pneumoniae* pneumonia

*Mycoplasma pneumoniae* pneumonia exhibits varying degrees of severity and is categorized into General *M. pneumoniae* Pneumonia (GMPP) for milder cases and Severe *M. pneumoniae* Pneumonia (SMPP) for more severe presentations (Zhang et al., 2022). Diagnosis of MPP was conducted following the Chinese Medical Association guidelines for management of CAP in children: (1) fever (>37.3°C) or acute respiratory symptoms or both; (2) decreased breath sounds, dry or wet rales; (3) chest radiograph showed at least one of the following: spotted or patchy infiltration; interstitial changes; lobar parenchymal infiltration shadow; enlargement of hilar lymph nodes; (4) positive PCR results or ≥4-fold increase in MP antibody titer. SMPP was defined as MPP with one of the following: (1) poor general condition; (2) increased breathing rate; (3) cyanosis and dyspnea; (4) multilobed or ≥2/3 infiltration of the lung; (5) transcutaneous oxygen saturation ≤92% in room air; (6) extrapulmonary complications.

### Classification of COVID-19 pandemic phases

Based on local interventions in Suzhou, the COVID-19 pandemic was categorized into four phases. In Phase I (March to May 2020), extensive NPIs such as school closures and mobility restrictions were implemented. During Phase II (June to August 2020), schools initiated a gradual reopening, prioritizing graduating classes, and kindergartens also partially resumed operations. The III phase (September 2020 to November 2022) featured the “Dynamic Zero-COVID” strategy, with widespread SARS-CoV-2 testing and responsive NPIs following any detected local transmission. Schools and kindergartens remained fully operational throughout this period. Finally, during Phase IV (January to November 2023), all NPIs were lifted.

### Statistical analysis

Continuous variables are presented as median (interquartile range, IQR), while categorical variables are presented as frequencies (percentages). Comparisons among different groups were made using the chi-square (*χ*^2^) test or Fisher’s exact test. Phylogenetic trees were constructed using R language and specific software packages. A two-sided *p*-value below 0.05 was considered statistically significant. Data were analyzed using SPSS 23.0 (released in 2015, SPSS Inc., Chicago, IL, United States).

## Results

From January 2016 to December 2023, this study included a total of 113,625 respiratory infection specimens from children across three centers. In the three centers, the *M. pneumoniae* positive detection rate was 21.1% before the pandemic. During the pandemic, the rates were 13.45% in Phase I, 4.9% in Phase II, and 7.8% in Phase III. After the pandemic, the rate in Phase IV rose to 45.3%.

We conducted a detailed analysis of the data from the Children’s Hospital of Soochow University. From January 2016 to December 2023, a total of 82,427 respiratory infection specimens from children were included in this study. The average monthly count of *M. pneumoniae*-positive cases was about 194 before the pandemic. This number fell to 37 cases per month in the pandemic. Post-pandemic, the average monthly count of *M. pneumoniae*-positive cases surged to 484.

**Figure 1:**
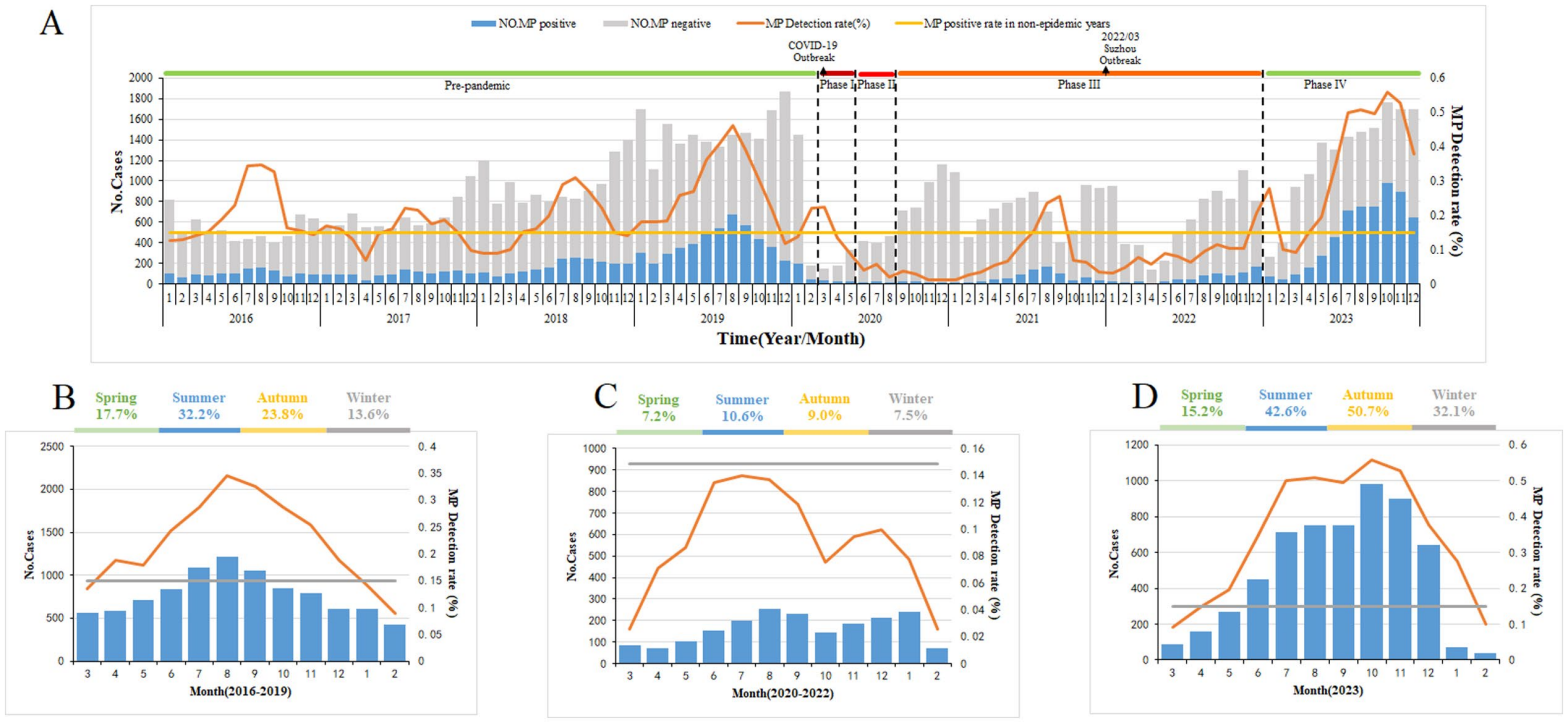
*Mycoplasma pneumoniae* (MP) prevalence among children with respiratory tract infection in Suzhou, China, from January 1, 2016, to December 31, 2023. **(A)** From January 1, 2016, to December 31, 2023, a total of 82,427 children with respiratory infections were tested for MP DNA, and the number of patients with respiratory tract infection and MP positive detection rates by month. **(B–D)** The average percentage of positive detections for MP among children with respiratory tract infections varies by season. Figure each month during the pre-epidemic **(B)**, epidemic **(C)**, and post-epidemic **(D)** periods. The gray bars **(A)** denote the number of MP-negative cases. The blue bars represent the MP-positive cases. The broken lines indicate the percentage of MP-positive cases among the total cases included in this study. The straight lines denote the threshold of MP-positive rate (14.89%) assigned in this study.

From 2016 to 2019, the annual positive detection rates for *M. pneumoniae* among children with respiratory infections were 19.26, 15.38, 17.56, and 27.22%, respectively. During the COVID-19 pandemic from 2020 to 2022, these rates notably dropped to 6.28, 8.60, and 9.56%, respectively. Then, in the post-pandemic year of 2023, the *M. pneumoniae* positive detection rate rose sharply to 39% (Figure 1A).

### Seasonality

In this study, the *M. pneumoniae* epidemic season was defined as consecutive months with a PCR positivity rate for *M. pneumoniae* exceeding 14.89%. Prior to the pandemic, *M. pneumoniae* epidemics typically began increasing in May and June, reaching a peak in August, and lasting about 7 months. In Phases I–III of the pandemic, the positive detection rate for *M. pneumoniae* was low. Nonetheless, in Phase IV, post-pandemic, the incidence of *M. pneumoniae* rebounded to endemic levels, featuring an epidemic season similar in timing but with a notably higher positive detection rate (Figures 1B–D).

### Positive detection rates of *M. pneumoniae* by age group and disease severity

**Figure 2:**
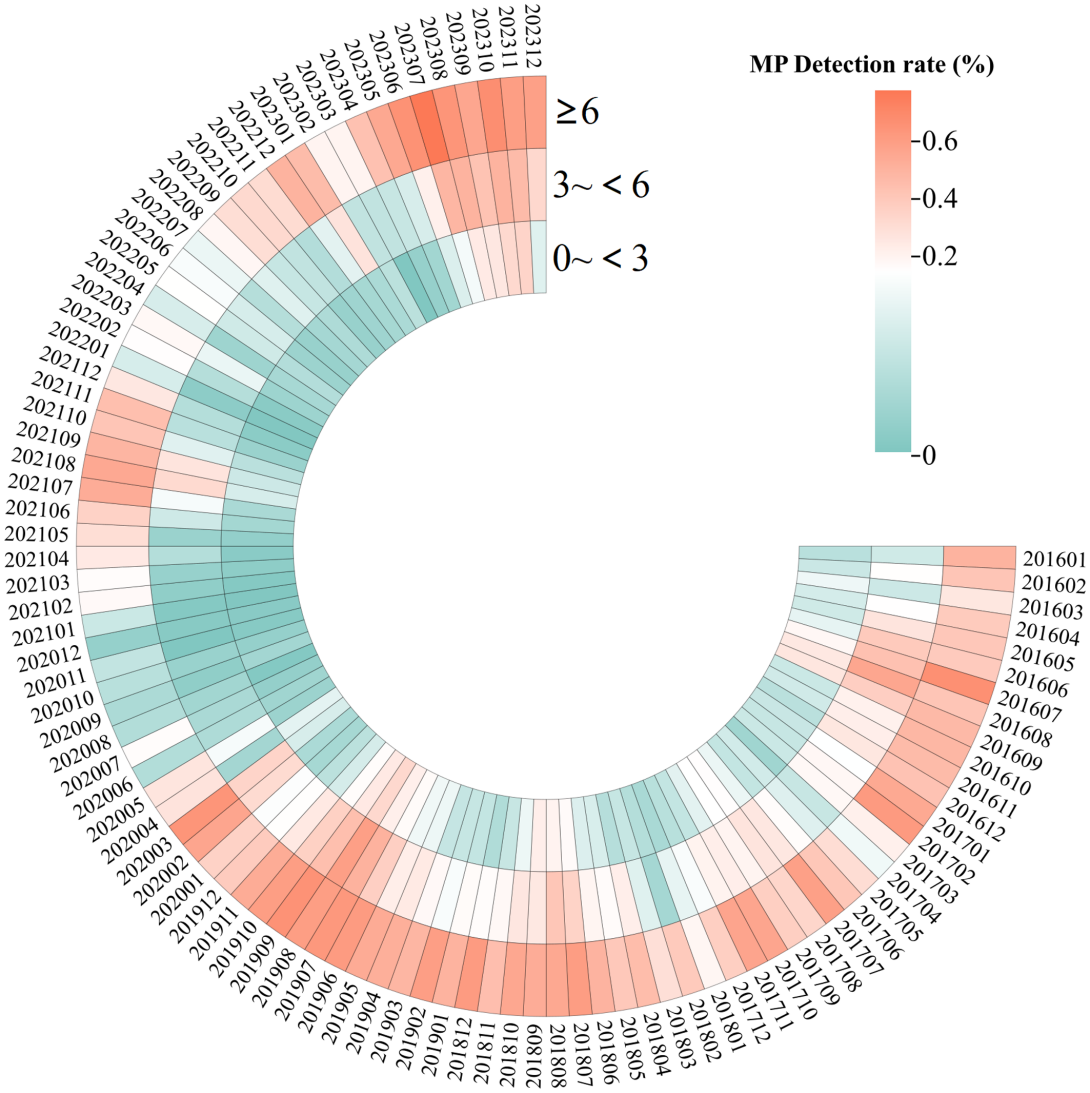
Heatmap displaying the detection rates of MP in 82,427 children with respiratory infections, from January 2016 to December 2023, with data categorized by age. The color intensity increases with higher detection rates, with red indicating the highest rates.

This study identified 2019 and 2023 as the peak years for *M. pneumoniae* positive detection rates. The age composition ratio in 2023 revealed a significantly higher proportion of children aged 6 and older compared with 2019. In 2019, 42.3% of cases involved children aged 6 years and older; by 2023, this proportion had significantly increased to 55.0%, indicating a statistically significant shift in age distribution (*χ*^2^ = 36.095, *p* < 0.001) (Figure 2).

**Figure 3:**
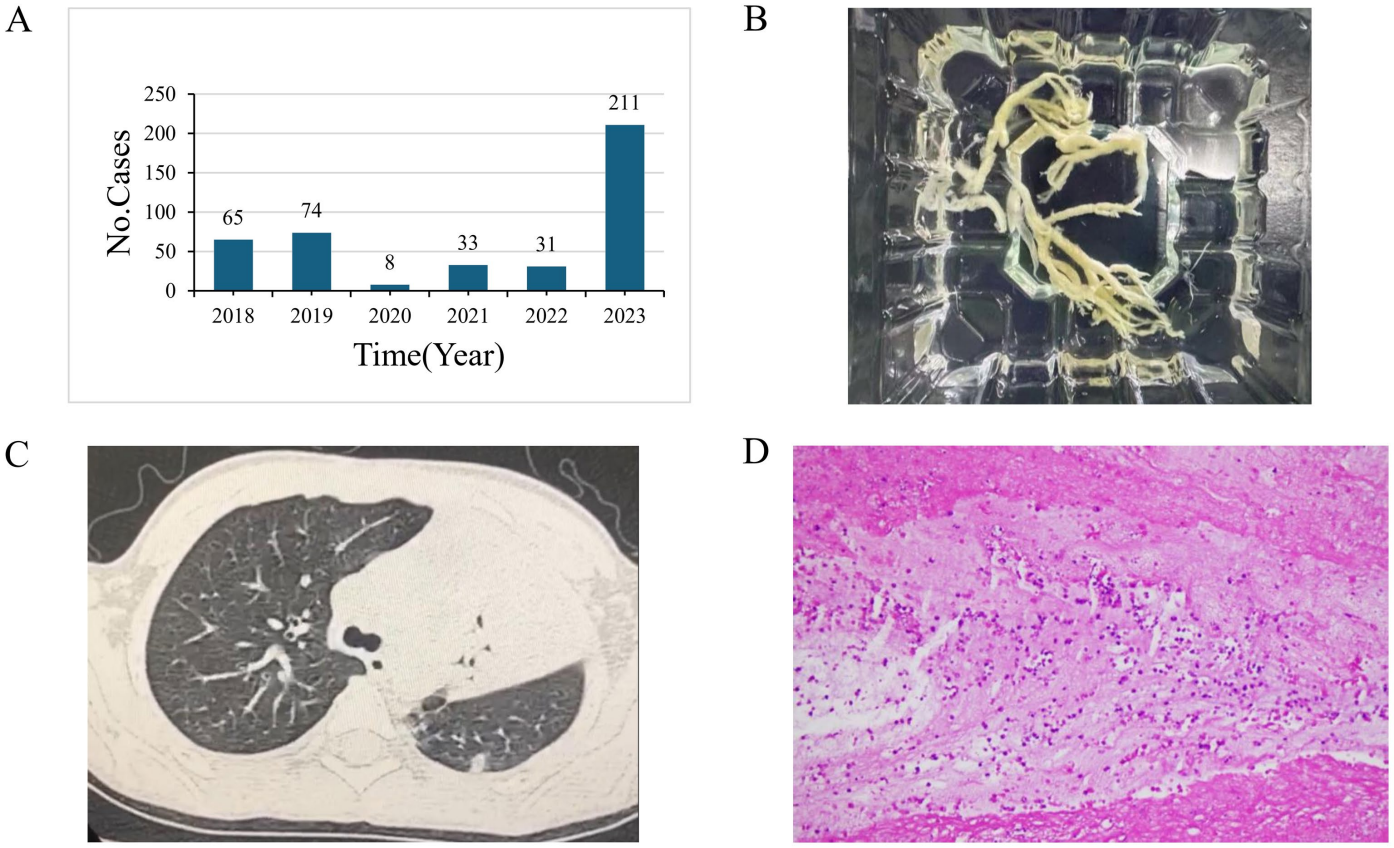
**(A)** The number of Plastic bronchitis (PB) cases from 2018 to 2023. **(B)** A tree-shaped sputum plug post-removal. **(C)** Chest CT findings in patients diagnosed with PB. **(D)** Pathological image from a tracheoscopic airway mucosal biopsy of a patient with PB, showing fibrin-like inflammatory exudates and a locally high lymphocytic and neutrophilic infiltrate.

Plastic bronchitis (PB) is a significant indicator of severe and refractory *M. pneumoniae* pneumonia. From 2018 to 2023, the annual counts of PB cases were 65, 74, 8, 33, 31, and 211, respectively, showing a marked increase in severity related to *M. pneumoniae* infection in 2023 (Figure 3A,B). PB on imaging often manifests as pulmonary consolidation and atelectasis (Figure 3C). The pathology of *M. pneumoniae* pneumonia frequently shows fibrinoid inflammatory changes (Figure 3D).

### Genotype and macrolide resistance mutations

In this study, 200 *M. pneumoniae*-positive cases from 2023 were randomly selected for P1 and SNP genotype analysis. Of the 200 samples, 8 were either suspected to have a low *M. pneumoniae* pathogen load. The remaining 192 samples were classified into 11 types based on the reference typing scheme, with the most common being SNP-27 (36.5%), SNP-0 (21.4%), SNP-11 (18.8%), and SNP-34 (17.7%). Further details are provided in Supplementary Table S2.

Among the 156 qualified BALF samples, 81.4% were P1 genotype and 18.6% were P2 genotype. Patients with severe *M. pneumoniae* pneumonia who had the P1 genotype were significantly more common than those with the P2 genotype (*p* = 0.021). There was no significant difference in the SNP genotype distribution between patients with GMPP and SMPP (*p* > 0.05) (Table 1).

**Table 1:** Characteristics comparison between severe *Mycoplasma pneumoniae* pneumonia (SMPP) and general *Mycoplasma pneumoniae* pneumonia (GMPP).

	SMPP (*N* = 77)	GMPP (*N* = 115)	*P-*value
SNP type			0.576
SNP0	21 (18.3%)	20 (26.00%)	
SNP1	2 (1.70%)	0 (0%)	
SNP2	1 (0.90%)	0 (0.00%)	
SNP3	3 (2.60%)	2 (2.60%)	
SNP5	1 (0.90%)	0 (0.00%)	
SNP10	1 (0.90%)	0 (0.00%)	
SNP11	23 (20.00%)	13 (16.90%)	
SNP15	22 (19.10%)	11 (14.30%)	
SNP16	0 (0.00%)	1 (1.30%)	
SNP18	0 (0.00%)	1 (1.30%)	
SNP27	41 (35.70%)	29 (37.70%)	
Macrolide-resistant			0.001
Positive	12 (6.5)	173 (93.5)	
Negative	0	7 (100)	
P1 genotype^a^			0.021
Type 1	32 (20.5%)	83 (66.9%)	
Type 2	10 (6.4%)	19 (12.1)	

The distribution of SNP subgroups is detailed in Supplementary Table S1, which offers a comprehensive breakdown of the genotypic variations observed. ^a^Of the 200 MP-positive BALF samples, 156 were qualified for P1 typing.

Of the 192 qualified BALF specimens analyzed for macrolide resistance mutations, the total mutation rate was 97.3%. The mutation rate for 23S rRNA at position 2063 was 96.17%, with A2063G being the most common mutation. Two samples had co-occurring mutations of A2063G and C2617T. The mutation rate at position 2064 in the 23S rRNA resistance gene was 1.6%. The mutation rate at the 2,617 site of the 23S rRNA was 1.0%.

**Figure 4:**
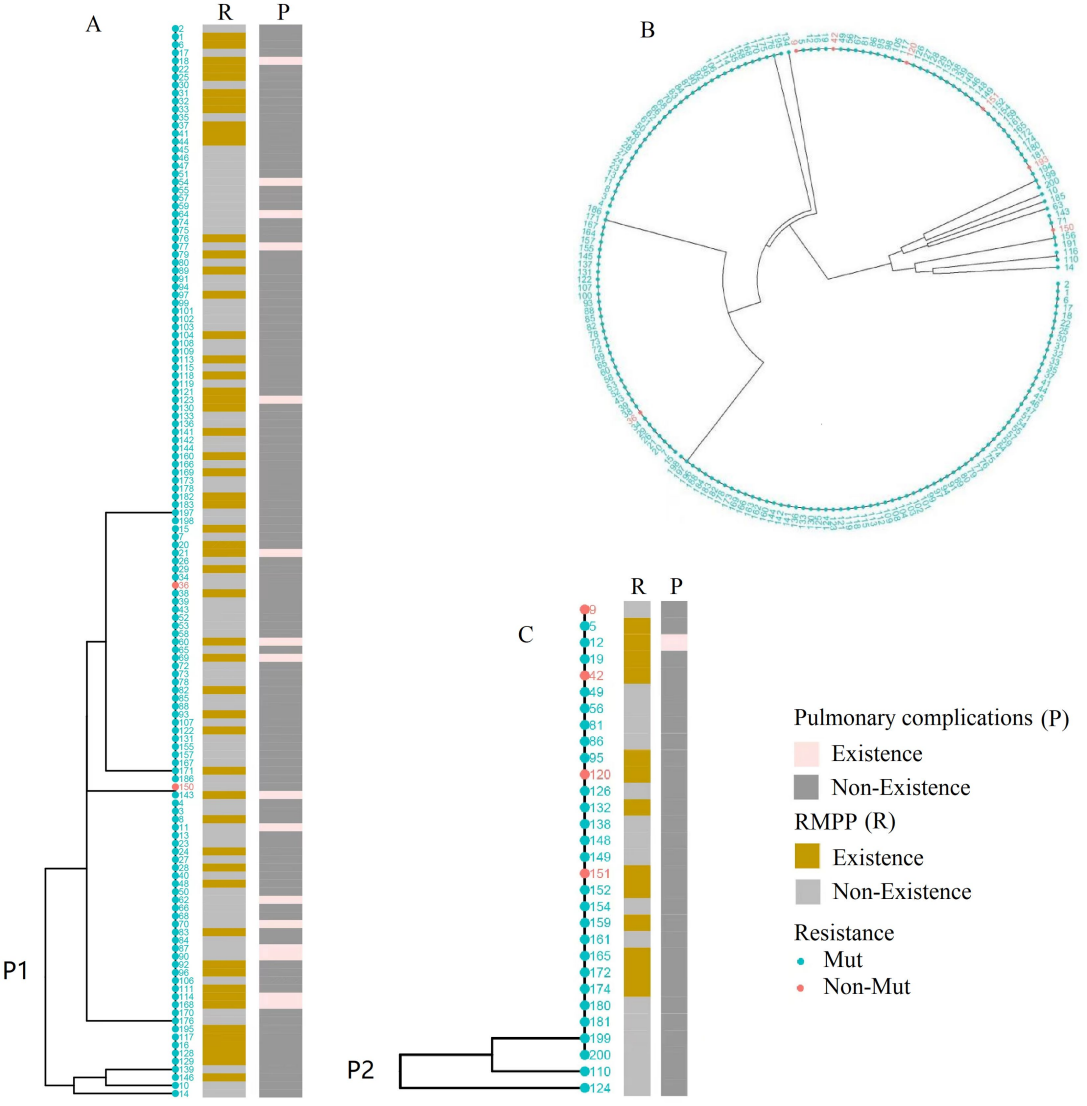
Phylogenetic analysis of *Mycoplasma pneumoniae* genotypes PI and SNP. **(A)** Phylogenetic tree based on genomic data from 127 P1 type 1 lineage strains. **(B)** Cluster analysis of 192 SNP-typed cases. strains. **(C)** Phylogenetic tree based on genomic data from 29 P1 type 2 lineage strains. The dots in the figures correspond to patient identification codes.

For the construction of the phylogenetic tree with the 156 *M. pneumoniae* positive cases, they were classified into P1 and P2 types based on P1 genotyping. The results indicated that the P1 type was predominant, with a significantly higher positive rate of macrolide resistance genes (*p* = 0.021) (Figures 4A,C). Cluster analysis of the 192 *M. pneumoniae* positive cases revealed no significant difference in macrolide resistance among different SNP types (Figure 4B).

## Discussion

In our current study, we utilized a multicenter, hospital-based epidemiological, clinical, and molecular methods to investigate changes in the epidemiological patterns of *M. pneumoniae* during various phases of the COVID-19 pandemic. We observed significant differences in the epidemiological patterns of *M. pneumoniae* among the population compared to pre-pandemic levels.

### Trends in *M. pneumoniae* infection in children

*Mycoplasma pneumoniae*, a ubiquitous pathogen with a global distribution, is responsible for 10–30% of CAP cases in children. Infections can lead to regional outbreaks every 3–5 years, lasting 1–2 years (Yamazaki and Kenri, 2016). Since the onset of the COVID-19 pandemic in Suzhou, the transmission trajectory of *M. pneumoniae* has been unusual, differing from previous epidemic patterns.

Our survey reveals that *M. pneumoniae* infections peaked in 2019 and again in 2023 within the period from 2016 to 2023. The incidence of *M. pneumoniae* pneumonia in 2023 significantly exceeds that of 2019, indicating a major outbreak of concern. Throughout Phases I–III of NPIs, *M. pneumoniae* positive cases were consistently below 200 per month, with a positivity rate lower than the average of 14.3%. The surge might be due to interventions such as mandatory mask-wearing and social distancing, or to antigenic shifts and increased population susceptibility (Chow et al., 2023; Liu B. et al., 2024). Herd immunity waned over the three-year NPIs period, due to inadequate exposure to the pathogen. Consequently, a weakened immune system struggles to combat *M. pneumoniae* infections following the relaxation of epidemic controls. This phenomenon is not unique to *M. pneumoniae*; it is also observed with other respiratory pathogens, such as influenza and respiratory syncytial virus (Jiang et al., 2024).

### Seasonality

*Mycoplasma pneumoniae* infections show a clear seasonal pattern. This study found that before the epidemic, peak infection rates were in summer, followed by autumn, while the lowest rates were in spring and winter (Tian et al., 2017; Wang et al., 2022). This finding is corroborated by earlier studies. Post-epidemic, autumn experienced the highest infection rates, with summer in the second place. Studies have established that *M. pneumoniae* optimally grows at 36–37°C, with infection rates rising as minimum temperatures increase. The warmer conditions of summer and autumn facilitate *M. pneumoniae* replication (Meyer Sauteur et al., 2022a,b). During the NPIs, strict control measures consistently kept *M. pneumoniae* positivity rates low, masking seasonal patterns. However, as these measures were relaxed, the seasonality of *M. pneumoniae* infections became more apparent. Related to the immune gap, the peak season’s outbreak appears delayed. The delayed reemergence of *M. pneumoniae* is likely due to waning herd immunity, as indicated by epidemic patterns. This delay is similar to the pattern observed with respiratory syncytial virus (RSV) (Li M. et al., 2024).

### Age

The positive detection rate of *M. pneumoniae* varied among different age groups in this study, with the school-age group having the highest rate, while the preschool, infant, and young children groups had the lowest. This finding is consistent with previous studies (Jiang et al., 2023). The elevated *M. pneumoniae* detection rate among school-age children could stem from decreased social distancing and heightened cross-infection following the relaxation of post-epidemic (Phase IV) control measures.

Further analysis of the relationship between *M. pneumoniae* positive detection rates and age across various phases showed that susceptibility to *M. pneumoniae* was lower in all age groups during the NPIs phase. Conversely, there was a significant increase in the percentage of positive *M. pneumoniae* detections in the age group above 6 years post-outbreak, and this difference was statistically significant.

This consideration is relevant to the prolonged implementation of NPIs, spanning up to 3 years. During the NPIs implementation, preschool and school-age children faced chronic masking, home isolation, reduced pathogen exposure, and weakened herd immunity. The phenomenon of mass infections following an outbreak is known as “immunization debt.” Similar observations regarding RSV infections were reported by Chinese scholar Jiang, who noted a significant shift in the age distribution of RSV infections in the post-epidemic period (Jiang et al., 2024).

### Counts of severe infections and ICU admissions

The PB is a relatively rare respiratory disease in children, characterized by the formation of gelatinous or firm bronchial casts within the airways. These casts can rapidly move along the bronchial tree, obstructing the airways and causing clinical symptoms such as shortness of breath and wheezing, and can be life-threatening in severe cases. PB is a critical complication of CAP, indicating severe disease. In our study, we quantified cases of PB diagnosed via bronchoscopy in patients testing positive for *M. pneumoniae* between 2018 and 2023.A notable upsurge in PB cases was observed in 2023.The pneumonia caused by *M. pneumoniae* infection in 2023 not only showed a significant rise in case numbers but also increased in severity, resulting in a considerable societal burden. The sudden increase in severe cases could be attributed to several factors. Initially, the risk of future epidemics increases as the proportion of susceptible individuals grows due to prolonged minimal exposure to pathogens. Notably, children born during the epidemic are especially impacted by the issue of immunization debt (Cohen et al., 2021). Our recent research indicates that prolonged minimal exposure to *M. pneumoniae* can weaken antibodies, highlighting an immune gap with attenuated humoral immunity as a key driver of increased *M. pneumoniae* susceptibility.

Secondly, macrolide antibiotics, commonly used to treat *M. pneumoniae*, have led to pathogen resistance due to overuse. Studies show that the resistance rate of *M. pneumoniae* to macrolides in China was between 70 and 90% before NPIs (Xue et al., 2018). This study revealed that the drug resistance rate of *M. pneumoniae* soared to 97.3% post-NPIs. The exceedingly high resistance rate to macrolides presents significant clinical challenges, underscoring the urgent need for alternative antibiotics to treat *M. pneumoniae* infections.

### Genotypes

*Mycoplasma pneumoniae* is divided into two main genotypes, P1 type 1 and P1 type 2, which are differentiated by nucleotide variations in the repetitive elements RepMP2/3 and RepMP4 of the MPN141 gene, encoding the P1 adhesin protein (Cousin-Allery et al., 2000). This study found that the P1 type 1 genotype was predominant in 2023. P1 type 1 showed a higher prevalence of severe infections, positive drug-resistant genes, and pulmonary complications than P1 type 2. This finding is consistent with previous research (Rodman et al., 2021). Consider genotypes as contributing factors to the prevalence of *M. pneumoniae* infections.

The increased accessibility of markers through high-throughput whole genome sequencing (WGS) has demonstrated SNP analysis’s advantages in assessing drug resistance, evolution, and molecular epidemiology (Lipworth et al., 2019). However, few clinical studies have investigated the influence of the *M. pneumoniae* SNP genotype to date. For genotype I, *M. pneumoniae* strains were predominantly SNP27, SNP11, and SNP15 types; for genotype II, SNP 0 was predominant in this study (Supplementary Table S1). Compared to the data from 2017 to 2018, there was a significant change in the genotype; SNP 14 was predominant in Suzhou during that period (Zhao et al., 2019).

### Limitations

This study did not comprehensively evaluate all environmental and behavioral factors that might influence the epidemiological patterns of *M. pneumoniae*, and thus has some limitations. Future studies should consider incorporating a broader spectrum of environmental and behavioral factors and examine their relationship with NPIs to thoroughly assess the epidemiological patterns of *M. pneumoniae*.

## Conclusion

Our study reveals significant shifts in the epidemiology of *M. pneumoniae* infections post-COVID-19 in Suzhou, China. The pandemic’s NPIs initially lowered *M. pneumoniae* incidence but were followed by a surge in cases. The resurgence underscores the need for ongoing vigilance and adaptive strategies to mitigate future *M. pneumoniae* outbreaks.

## Data Availability

The original contributions presented in the study are included in the article/supplementary material, further inquiries can be directed to the corresponding author/s.
