# Supplementary material for: Epidemiological, clinical, and genotypic characteristics of pediatric Mycoplasma pneumoniae infections: an 8-year survey in Suzhou, China in the pre- and post-COVID-19 eras
Source: Front Microbiol. 2024 Oct 15;15:1483152. doi: 10.3389/fmicb.2024.1483152 (PMC11518825; doi:10.3389/fmicb.2024.1483152)
Supplement: Supplementary file 1 [file Data_Sheet_1.pdf]

Supplementary materials

Table 1S. Overview of SNP profiles for six selected SNP markers in the gene of MP .

P1 gene type		SNP sites	SNP name[a]						Aligned result of SNPs
			MPN114-1461	MPN126-470	MPN213-47	MPN262-192	MPN280-1641	MPN372-1112	
	MLVA	Type of SNP	C/T	C/T	G/T	C/T	G/T	G/T	
2	3562	SNP 0	C	C	G	C	G	G	000000
1	4572	SNP 1	C	C	G	C	G	T	000001
2	-	SNP 2	C	C	G	C	T	G	000010
1	4572	SNP 3	C	C	G	C	T	T	000011
1	-	SNP 5	C	C	G	T	G	T	000101
2	-	SNP 10	C	C	T	C	T	G	001010
1	4572	SNP 11	C	C	T	C	T	T	001011
1	4573	SNP 15	C	C	T	T	T	T	001111
2	-	SNP 16	C	T	G	C	G	G	010000
2	-	SNP 18	C	T	G	C	T	G	010010
1	4572	SNP 27	C	T	T	C	T	T	011011

[a]The SNP name corresponds to the name or the mnemonic of the gene containing the SNP and the position of the SNP in the gene of MP. The number after MPN is the gene number, and the superscript number indicate the position of SNP in the gene.

Data analysis of gene

The SNP sites of 6 genotypes were aligned based on the MPN gene (i.e., MPN114, 126, 213, 262, 280, 372). For the SNPs, A/T was assigned a value of 1, and C/G was assigned a value of 0. On this basis, each SNP genotyped isolate was represented by six numbers consisting of "0" or "1" from 000000 to 111111. This SNP number was transformed into a decimal scale of 0-63 according to the binary system, yielding 64 genotypes in total. For the M129 strain containing 6 SNP sites of C/C/G/C/G/T, the number was 000001, which resulted in a score of 1 after transformation into a binary digit. Therefore, the M129 strain was defined as the SNP1 type. See Table 1S for specific SNP typing principles.

Primer and Probe Details for *M. pneumoniae* SNP Genotyping Assay

Primer Sequences for M. pneumoniae:

MPN1141461

Forward Primer: 5'-acgttggatgCA-3'

Reverse Primer: 5'-CCGAGTGTC-3'

MPN126470

Forward Primer: 5'-acgttggatgAA-3'

Reverse Primer: 5'-ATTCCCCGT-3'

MPN21347

Forward Primer: 5'-acgttggatgA-3'

Reverse Primer: 5'-TCAGTCGCT-3'

MPN262192

Forward Primer: 5'-acgttggatgAC-3'

Reverse Primer: 5'-CACTTAGAG-3'

MPN2801641

Forward Primer: 5'-acgttggatgCTC-3'

Reverse Primer: 5'-AATTAAACGCG-3'

MPN3721112

Forward Primer: 5'-acgttggatgAGTG-3'

Reverse Primer: 5'-TTAGCGCGGTT-3'

Probe Design:

23S rRNA2063

Probe Sequence: 5'-AAGACAC-3'

Mass Probe Mass (Da): 5589.6 (G), 5902.6 (A), 5886.6 (C), 5862.6 (T)

23S rRNA2064

Probe Sequence: 5'-AAGACAC-3'

Mass Probe Mass (Da): 7995.2 (T), 8337.2 (C), 8268.2 (G), 8308.2 (A)

23S rRNA2617

Probe Sequence: 5'-TTAAAGAG-3'

Mass Probe Mass (Da): 6478.2 (C), 6751.2 (G), 6791.2 (A), 6775.2 (T)

The probe design is integrated with the PCR products through Single Base Extension (SBE) technology, utilizing specific mass probes to identify and distinguish different SNP sites.

Table 2S. Distribution of SNP genotypes in this study

Type of SNP	0	1	2	3	5	10	11	15	16	18	27
Number of samples	41	2	1	5	1	1	36	34	1	1	70

200 MP positives in BALF samples have eight samples for suspected negative or low pathogenic loads (code ID: 147, 153, 177, 179, 187, 189, 190, 192).The rest of the 192 samples according to the reference classification scheme can be divided into 11 kinds of classification (Table2S).
